# Socio-economic factors do not affect overall survival in soft tissue sarcoma when patients treated at a single high-volume center

**DOI:** 10.1186/s12885-021-08352-z

**Published:** 2021-05-27

**Authors:** Boryana M. Eastman, Daniel S. Hippe, Landon S. Wootton, Matthew J. Nyflot, Matthew J. Thompson, Seth M. Pollack, Edward Kim, Matthew B. Spraker

**Affiliations:** 1 Department of Radiation Oncology, University of Washington School of Medicine, 1959 NE Pacific St, Seattle, WA 98195 USA; 2 Department of Radiology, University of Washington School of Medicine, Seattle, WA 98195 USA; 3 Department of Radiation Oncology, Baylor Scott and White Cancer Center, Round Rock, TX 78665 USA; 4 Department of Orthopedics and Sports Medicine, University of Washington School of Medicine, Seattle, WA 98195 USA; 5 Department of Medicine (Hematology and Oncology), Northwestern University Feinberg School of Medicine, Chicago, IL 60611 USA; 6 Department of Radiation Oncology, Washington University in Saint Louis, St. Louis, MO 63130 USA

**Keywords:** Sarcoma, Socio-economic factors, Disparity, Radiation, High-volume center

## Abstract

**Background:**

Treatments for soft tissue sarcoma (STS) include extensive surgical resection, radiation and chemotherapy, and can necessitate specialized care and excellent social support. Studies have demonstrated that socioeconomic factors, such as income, marital status, urban/rural residence, and educational attainment as well as treatment at high-volume institution may be associated with overall survival (OS) in STS.

**Methods:**

In order to explore the effect of socio-economic factors on OS in patients treated at a high-volume center, we performed a retrospective analysis of STS patients treated at a single institution.

**Results:**

Overall, 435 patients were included. Thirty-seven percent had grade 3 tumors and 44% had disease larger than 5 cm. Patients were most commonly privately insured (38%), married (67%) and retired or unemployed (43%). Median distance from the treatment center was 42 miles and median area deprivation index (ADI) was 5 (10 representing most deprived communities). The majority of patients (52%) were treated with neoadjuvant therapy followed by resection. As expected, higher tumor grade (HR 3.1), tumor size > 5 cm (HR 1.3), and involved lymph nodes (HR 3.2) were significantly associated with OS on multivariate analysis. Demographic and socioeconomic factors, including sex, age at diagnosis, marital status, employment status, urban vs. rural location, income, education, distance to the treatment center, and ADI were not associated with OS.

**Conclusions:**

In contrast to prior studies, we did not identify a significant association between socioeconomic factors and OS of patients with STS when patients were treated at a single high-volume center. Treatment at a high volume institution may mitigate the importance of socio-economic factors in the OS of STS.

**Supplementary Information:**

The online version contains supplementary material available at 10.1186/s12885-021-08352-z.

## Background

Soft tissue sarcomas (STS) represent a heterogenous group of rare, aggressive tumors accounting for 0.5% of all malignancies and over 5000 annual deaths in the United States alone [1]. Multiple clinical and tumor factors have been associated with adverse outcomes, including older age, larger tumor size, higher grade, histology type and the presence of loco-regional spread or distant metastasis [2, 3].

More recently, a growing body of evidence has also suggested that the case volume of treatment centers may influence treatment outcomes. For example, patients with STS who were treated at high volume centers had improved overall survival compared to patients treated at centers with lower volumes [4, 5]. This may be due to the fact that many sarcomas require complex multimodality therapy including surgical resection, chemotherapy, and advanced radiation therapy, which may require experience and special expertise [6–9]. Alternatively, high-volume centers may unintentionally select for patients more likely to have favorable outcomes, possibly in part due to socioeconomic factors. Prior authors have discussed that such retrospective studies must be interpreted with caution [10].

Indeed, numerous studies have demonstrated that certain socio-economic factors are associated with clinical outcomes of patients with multiple cancer types, including STS [11]. For example, studies have shown that patients with STS who are married [12, 13], have private insurance [14], are white [15, 16], or live in an urban setting [15] have better overall survival compared to patients who are not in these groups. This trend is also present in Europe, where studies have revealed that patients with lower income, lower educational attainment or higher deprivation captured by a composite index also have worse overall survival [17–19]. However, most of these studies have examined large cancer registries, such as the United States National Cancer Database (NCDB) or the surveillance, epidemiology, and end results (SEER) database. Such databases lack important clinical, treatment, and tumor factors, which can confound interpretation of the association between socio-economic factors and clinical outcomes [20].

We therefore developed a detailed database including clinical, tumor, and socio-economic factors and outcomes. We sought to explore the association between socio-economic factors and overall survival for patients with STS treated at a high volume academic center with the hypothesis that socio-economic factors may play less of a role in disease outcomes when patients receive specialized multimodality care at a single institution. These data provide important first steps to understand how sarcoma patients’ outcomes are related to socioeconomic factors, and to guide us toward targeted quality improvement for this patient population.

## Methods

### Patient identification

A retrospective chart review of patients with newly diagnosed, pathologically-confirmed STS who were treated at a single large academic center, University of Washington Medical Center, between 1990 and 2016 was performed. Patients older than 18 years of age who lived in the state of Washington were included in the analysis. Patients with metastatic disease at diagnosis were excluded. This study was approved by the local Institutional Review Board and all methods were carried out in accordance with relevant guidelines and regulations. Informed consent was waived by the Human Subjects Division, Committee D, Institutional Review Board at the University of Washington.

### Clinical and socioeconomic characteristics

Clinical stage, Federation Nationale des Centres de Lutte Contre Le Cancer (FNCLCC) grade, treatment modality, permanent residence ZIP code, marital status, employment status, and insurance status were obtained from the institutional electronic medical record. Average income and average educational attainment were obtained from the US Census bureau American Fact Finder for each patient’s permanent residence ZIP code (US Census). Educational attainment was separated in quartiles with Q1 representing the top 25% of ZIP codes as ordered by highest percentage of the population obtaining a college degree or higher. Rural-Urban Code (RUC) classifications of each ZIP code were obtained from the United States Department of Agriculture Electronic Research Service. Areas with RUC codes of 1–3 were defined as metro areas and RUC 4–9 were defined as non-metro areas per the US Department of Agriculture (https://www.ers.usda.gov/data-products/rural-urban-continuum-codes/). State-specific Area Deprivation Index (ADI) deciles were obtained from the University of Wisconsin School of Medicine and Public Health and linked using permanent address ZIP code (https://www.neighborhoodatlas.medicine.wisc.edu/). ADI is a composite measure of neighborhood socioeconomic disadvantage that uses 17 census measures capturing education, employment, income, poverty, and housing characteristics [21]. ADI ranges from 1 to 10 with 10 considered the most deprived. Distance from permanent address ZIP code to treatment facility in miles was calculated in Google Maps using the function googleapis.

### Statistical analysis

Clinical and socio-economic variables were compared between groups using Fisher’s exact test or the Wilcoxon rank-sum test. Spearman’s rank correlation coefficient was used to evaluate the strength of relationship between inter-related socio-economic variables: income, education, distance from treatment facility, urban/rural, and ADI. Overall survival (OS) was defined as the time from pathologic diagnosis to death from any cause, censored at the date of the last clinical record. OS was summarized using Kaplan-Meier curves. Relationships between clinical and socioeconomic variables and OS were evaluated using univariate and multivariate Cox regression models for all patients. Data was presented as regression coefficients reflecting the increase in risk for the described change (e.g. 10-year increase in age, 50% increase in average income, 1-decile increase in ADI). Since there is substantial variability in patient age and treatment paradigms with STS, analysis was additionally performed for these subgroups. All statistical calculations were conducted with the statistical computing language R (version 3.1.1; R Foundation for Statistical Computing, Vienna, Austria). Throughout, two-sided tests were used, with statistical significance defined as *p* < 0.05. No *p*-value adjustments were made for multiple comparisons. Multivariate analysis was performed on the entire cohort and the largest treatment subgroup treated with neoadjuvant multimodality therapy.

## Results

### Cohort characteristics

**Fig. 1:**
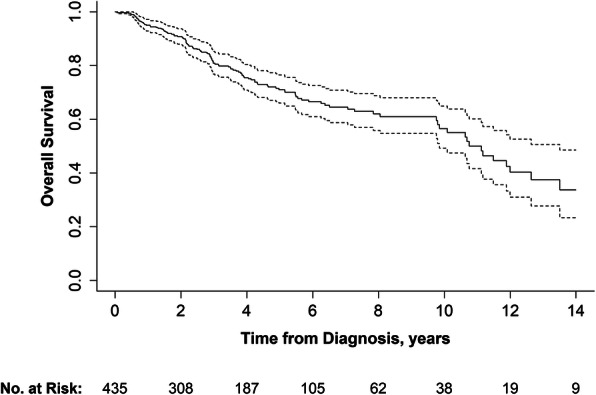
Kaplan-Meier estimate of overall survival. Dashed lines indicate the 95% confidence bands. Tick marks indicate censoring

**Fig. 2:**
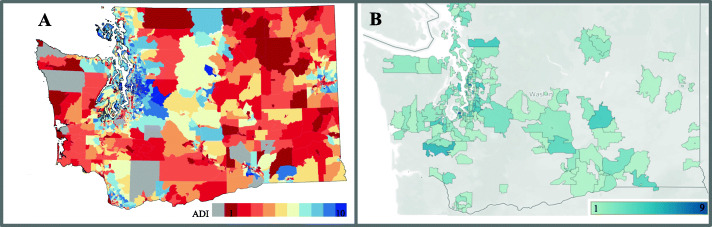
**a** Heat map of ADI distribution by ZIP code in Washington State (image obtained from University of Wisconsin School of Medicine and Public Health, https://www.neighborhoodatlas.medicine.wisc.edu/) **b** Heat map of number of patients per ZIP code, overlaid over map of Washington state (Tableau, Inc)

A total of 891 patients with STS were evaluated at our center over 27 years (average 33 patients/year) with 435 patients meeting the inclusion criteria. A consort diagram is shown in Supplemental Figure 1. After a median follow-up of 3 years (range 18 days – 18 years), a total of 120 deaths (28%) were observed with median survival of 11 years after diagnosis (Fig. 1). The median age at diagnosis was 53 years (range 18–92) and 103 patients (24%) were over the age of 65 (Supplemental Table 1). 44% of patients had tumors larger than 5 cm and 37% of patients had grade 3 tumors. Ten patients (2.3%) had node positive disease. In terms of socio-economic characteristics, 67% of patients were married or had a domestic partner, 36.8% were employed full time and 37.9% had private insurance. Mean average zip code income was $67,023 ± 20,458. Median distance from the treatment center for each patient was 42 miles (range 2–320). The patient population was predominantly urban with 59.5% of patients living in metropolitan areas (Fig. 2) and median ADI of 5 (range 1–10). As expected, average income, education, and distance from treatment center were strongly correlated with ADI (Supplemental Table 2). As such, these individual variables were not included in further multivariate analyses and only ADI was used.

### Treatment characteristics

**Fig. 3:**
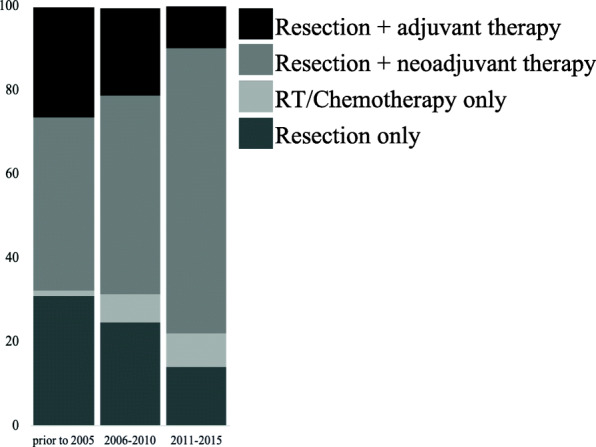
Distribution of treatment modalities per time period

Patients were treated with variety of modalities, with the majority of patients (51%) undergoing neoadjuvant therapy prior to resection. The remaining patients were treated with resection alone (19%), adjuvant therapy post resection (24%), or chemotherapy or radiation therapy alone (5%) (Table 1). Of note, sarcoma treatment strategies at our institution have evolved over the last 30 years with neoadjuvant treatment becoming more common recently. Between 2011 and 2015, 68% of patients were treated with neoadjuvant therapy, compared to 48% of patients treated between 2006 and 2010 and 41% of patients treated prior to 2005 (Fig. 3, *p* < 0.02 for all trends).

**Table 1:** Patient characteristics by treatment group (*N* = 435)

	All Patients	Treatment Type				
		Resection only (%) (***N*** = 84)	RT/Chemo Only (%) (***N*** = 23)	Resection + Neoadjuvant therapy (%) (***N*** = 225)	Resection + Adjuvant therapy (%) (***N*** = 103)	***P***-value *
**Demographics**						
Sex						0.60
Male	237 (54.5)	51 (60.7)	13 (56.5)	120 (53.3)	53 (51.5)	
Female	198 (45.5)	33 (39.3)	10 (43.5)	105 (46.7)	50 (48.5)	
Age at diagnosis, years (range)	53 (18–92)	57	57	51	53	**0.005**
Insurance status						**0.002**
Private	163 (37.9)	22 (26.2)	7 (30.4)	101 (44.9)	35 (34.0)	
Private + Medicare/Medicaid	20 (4.6)	2 (2.4)	0 (0.0)	15 (6.7)	3 (2.9)	
Medicare	12 (2.8)	2 (2.4)	0 (0.0)	5 (2.2)	5 (4.9)	
Medicaid	23 (5.3)	0 (0.0)	2 (8.7)	14 (6.2)	7 (6.8)	
Unknown	215 (49.4)	58 (69.0)	14 (60.9)	90 (40.0)	53 (51.5)	
Marital Status						0.25
Married/significant other	292 (67.1)	56 (66.7)	13 (56.6)	154 (68.4)	69 (67.0)	
Single/separated/divorced	140 (32.2)	27 (32.1)	9 (39.1)	71 (31.6)	33 (32.0)	
Unknown	3 (0.7)	1 (1.2)	1 (4.3)	0 (0.0)	1 (1.0)	
Employment status						**< 0.001**
Full time/part time/student	160 (36.8)	23 (27.4)	7 (30.4)	89 (39.6)	41 (39.8)	
Unemployed/retired	187 (43.0)	29 (34.5)	11 (47.8)	106 (47.1)	41 (39.8)	
Unknown	88 (20.2)	32 (38.1)	5 (21.70)	30 (13.3)	21 (20.4)	
Location						0.30
Metropolitan area (> 1 M)	259 (59.5)	45 (53.6)	12 (52.2)	137 (60.9)	65 (63.1)	
Metropolitan area (250 K-1 M)	63 (14.5)	13 (15.5)	5 (21.7)	26 (11.6)	19 (18.4)	
Metropolitan area (< 250 K)	51 (11.7)	15 (17.9)	3 (13.0)	27 (12.0)	6 (5.8)	
Non-metropolitan area	62 (14.3)	11 (13.1)	3 (13.0)	35 (15.6)	13 (12.6)	
Average income, $	67,023	59,741	63,944	61,895	66,435	0.087
Education quartile						0.34
Q1	40 (9.3)	11 (13.1)	2 (9.1)	23 (10.5)	4 (3.9)	
Q2	102 (23.8)	23 (27.4)	5 (22.7)	47 (21.5)	27 (26.2)	
Q3	114 (26.6)	17 (20.2)	4 (18.2)	66 (30.1)	27 (26.2)	
Q4	172 (40.2)	33 (39.3)	11 (50.0)	83 (37.9)	45 (43.7)	
Median distance, miles	42	46	43	43	42	0.76
ADI WA decile (range)	5 (1–10)	6	6	5	5	0.17
**Tumor Characteristics**						
Grade						**< 0.001**
1	61 (14.0)	24 (28.6)	1 (4.3)	16 (7.1)	20 (19.4)	
2	112 (25.7)	20 (23.8)	3 (13.0)	62 (27.6)	27 (26.2)	
3	161 (37.0)	18 (21.4)	13 (56.5)	102 (45.3)	28 (27.2)	
Unknown	101 (23.2)	22 (26.2)	6 (26.1)	45 (20.0)	28 (27.2)	
Maximum extent on imaging						**< 0.001**
≤ 5 cm	57 (13.1)	6 (7.1)	6 (26.1)	30 (13.3)	15 (14.6)	
> 5 cm	194 (44.8)	19 (22.6)	10 (43.5)	134 (59.6)	31 (30.1)	
Unknown	184 (42.3)	59 (70.2)	7 (30.4)	61 (27.1)	57 (55.3)	
Clinical N stage						0.069
N0	410 (94.3)	82 (97.6)	19 (82.6)	211 (93.8)	98 (95.1)	
N1	10 (2.3)	0 (0.0)	3 (13.0)	6 (2.7)	1 (1.0)	
Unknown	15 (3.4)	2 (2.4)	1 (4.3)	8 (3.6)	4 (3.9)	
**Treatment**						
Median time from diagnosis, days (range)	34 (2–240)	56 (2–240)	27 (4–108)	30 (3–178)	35 (6–188)	**< 0.001**

*Wald test of HR = 1, without adjustment for multiple comparisons

### Overall cohort treatment outcomes

The relationship between clinical, socio-economic factors, and OS was tested in an univariate analysis for all patients. Grade 3 disease (HR 3.34 vs. grade 1, CI 1.66–7.73) and N1 disease (HR 2.94 vs. N0, CI 1.19–7.24) were associated with worse OS. No other clinical or socio-economic factors were statistically significantly associated with OS in the entire cohort, though there were some trends with better survival in female patients (HR 0.71 vs. male, CI 0.49–1.02, *p* = 0.066) and patients who lived in rural areas (HR 0.55 vs. urban, CI 0.29–1.06, *p* = 0.073) or farther away from the treatment center (HR: 0.94 per 50%-increase in distance, CI 0.99–1.00, *p* = 0.057) (Table 2). Multivariate analysis accounting for all clinical variables, employment status (HR=1.18 for unemployed vs employed, *p* = 0.72), marital status (HR 0.94 for single vs. married, *p* = 0.76), and ADI (HR 0.95 per 1-decile increase, *p* = 0.16) did not reveal any significant association between socio-economic factors and OS.

**Table 2:** Univariate and multivariate analysis of predictors of overall survival in the entire patient cohort

	Univariate Analysis			Multivariate Analysis		
	HR	95% CI	***p***-value*	HR	95% CI	***p***-value*
**Variable**						
Female Sex	0.71	(0.49–1.02)	0.066	0.74	(0.51–1.08)	0.12
Age at diagnosis, per 10-year increase	1.09	(0.97–1.23)	0.15	1.06	(0.94–1.21)	0.33
Marital Status			0.96			0.76
Married/significant other	{ref.}			{ref.}		
Single/separated/divorced	1.01	(0.69–1.48)		0.94	(0.63–1.40)	
Employment status			0.32			0.72
Full time/part time/student	{ref.}			{ref.}		
Unemployed/retired	1.36	(0.91–2.01)		1.18	(0.77–1.80)	
Unknown	1.21	(0.69–2.13)		1.19	(0.66–2.16)	
Location			0.073			
Urban (Metropolitan)	{ref.}					
Rural (Non-metropolitan)	0.55	(0.29–1.06)				
Average income per 50% increase	1.12	(0.88–1.43)	0.36			
Education quartile per 1-quartile increase	1.03	(0.86–1.24)	0.73			
Mean distance per 50% increase	0.94	(0.88–1.00)	0.057			
ADI WA decile per 1-decile increase	0.97	(0.90–1.04)	0.39	0.95	(0.88–1.02)	0.16
**Tumor Characteristics**						
Grade			**< 0.001**			**< 0.001**
1	{ref.}			{ref.}		
2	1.39	(0.65–2.97)		1.43	(0.66–3.10)	
3	3.34	(1.66–6.73)		3.18	(1.54–6.54)	
Unknown	1.68	(0.77–3.65)		1.57	(0.72–3.45)	
Maximum extent on imaging			0.49			0.74
≤ 5 cm	{ref.}			{ref.}		
> 5 cm	1.46	(0.76–2.81)		1.29	(0.66–2.50)	
Unknown	1.46	(0.76–2.80)		1.29	(.066–2.53)	
Clinical N stage			**0.019**			**0.017**
N0	{ref.}			{ref.}		
N1	2.94	(1.19–7.24)		3.07	(1.22–7.71)	
Unknown				1.94	(0.89–4.24)	
**Treatment**						
Time from diagnosis to treatment, per 50% increase	0.93	(0.84, 1.04)	0.20	0.96	(0.86–1.09)	0.55

*Wald test of HR = 1, without adjustment for multiple comparisons

### Treatment paradigm subgroup analysis

A subgroup analysis of patients by treatment paradigm type (i.e. resection only, RT/chemo only, resection + neoadjuvant RT/chemotherapy, and resection + adjuvant RT/chemotherapy) was also performed (Table 1). The patients treated with multimodality therapy were significantly younger and more likely to be employed full time than patients treated with resection, RT, or chemotherapy alone (*p* < 0.005). Additionally, patients treated with neoadjuvant therapy had a higher proportion of tumors with higher grade (grade 2 and 3) and size > 5 cm, when compared to patients treated with resection alone or receiving adjuvant therapy (*p* < 0.001). Univariate analysis of clinical and socio-economic factors again identified nodal status and grade 2 and 3 disease as associated with worse OS (*p* < 0.05 in patients treated with resection only, RT/chemo only and resection + neoadjuvant RT/chemotherapy). In the group of patients treated with neoadjuvant therapy followed by resection, increasing distance was associated with better OS (HR: 0.91 per 50% increase in distance, *p* = 0.039).

Income, education, and distance were all inter-correlated and all three were correlated with ADI (Spearman’s rank correlation coefficient ρ 0.54–0.81) (Supplemental Table 2). Urban/rural classification was also correlated with ADI (ρ = 0.3) and rural classification corresponded strongly to ADI = 9 (Supplemental Figure 2). Given the correlation between these factors and its composite nature, only ADI was included in the multivariate analysis. The multivariate analysis did not reveal any further significant associations between socio-economic factors and OS, though there was a trend toward better survival with higher ADI (HR 0.91 per 1-decile increase, CI 0.82–1.01, *p* = 0.071 (Table 3). No other clinical or socio-economic factors were significantly associated with OS in any of the analyzed groups.

**Table 3:** Multivariate analysis of predictors of overall survival in the resection and neoadjuvant therapy group

	Resection + Neoadjuvant therapy (***N*** = 225)		
	HR	(95% CI)	***p***-value*
**Variable**			
Female sex	0.76	(0.45, 1.29)	0.31
Age at diagnosis, per 10-year increase	0.97	(0.81, 1.16)	0.74
Marital status			0.24
Married/significant other	{ref.}		
Single/separated/divorced	1.44	(0.78, 2.67)	
Employment status			0.13
Full time/part time/student	{ref.}		
Unemployed/retired	1.81	(0.97, 3.37)	
Unknown	2.04	(0.81, 5.14)	
ADI WA decile, per 1-decile increase	0.91	(0.82, 1.01)	0.071
**Tumor Characteristics**			
Grade			**0.018**
1	{ref.}		
2	1.62	(0.21, 12.78)	
3	4.45	(0.60, 33.17)	
Unknown	3.23	(0.41, 25.54)	
Maximum extent on imaging			0.99
≤ 5 cm	{ref.}		
> 5 cm	0.96	(0.43, 2.15)	
Unknown	0.95	(0.40, 2.29)	
**Treatment**			
Time from diagnosis to first treatment, per 50% increase	0.99	(0.80, 1.23)	0.95

*Wald test of HR = 1, without adjustment for multiple comparisons

### Patient age subgroup analysis

Older age (age ≥ 50 years old) has been generally associated with worse OS in a number of solid tumors including sarcoma, so we compared the OS of patients ≥50 years old to the younger adult population (Table 4) [22]. The median ages at diagnosis were 37 and 61 years for the younger and older populations, respectively (Table 4). Patients in the older group were less likely to be employed full time and were more likely to be married or have a domestic partner (Table 4). As was found in the total patient population, higher grade and nodal status were associated with worse OS in both the groups (*p* < 0.05). Among patients over the age of 50, women had a significantly lower risk of death (HR 0.51 vs. male, CI 0.30–0.87, *p* = 0.013). No other clinical or socio-economic factors were significantly associated with OS in the two subgroups (Table 5).

**Table 4:** Patient characteristics by age subgroup

	Age < 50 (***N*** = 173) (%)	Age ≥ 50 (***N*** = 262) (%)	***P***-value
**Demographics**			
Sex			0.84
Male	93 (53.8)	144 (55.0)	
Female	80 (46.2)	118 (45.0)	
Median age at diagnosis, years	37	61	
Insurance status			0.054
Private	71 (41.0)	94 (35.9)	
Private + Medicare/Medicaid	9 (5.2)	11 (4.2)	
Medicare	2 (1.2)	10 (3.8)	
Medicaid	14 (8.1)	9 (3.4)	
Unknown	77 (44.5)	138 (52.7)	
Marital status			**0.010**
Married/significant other	103 (59.5)	189 (72.1)	
Single/separated/divorced	68 (39.3)	72 (27.5)	
Unknown	2 (1.2)	1 (0.4)	
Employment status			**< 0.001**
Full time/part time/student	88 (50.9)	72 (27.5)	
Unemployed/retired	53 (30.6)	134 (51.1)	
Unknown	32 (18.5)	56 (21.4)	
Location			0.33
Metropolitan area (> 1 M)	112 (64.7)	147 (56.1)	
Metropolitan area (250 K-1 M)	21 (12.1)	42 (16.0)	
Metropolitan area (< 250 K)	17 (9.8)	34 (13.0)	
Non-metropolitan area	23 (13.3)	39 (14.9)	
Average income, $	63,217	62,575	0.54
Education quartile			0.86
Q1	18 (10.6)	22 (8.5)	
Q2	42 (24.7)	60 (23.3)	
Q3	44 (25.9)	70 (27.1)	
Q4	66 (38.8)	106 (41.1)	
Mean distance by zip code, miles	42	43	0.61
Area deprivation index (ADI) decile	6	5	0.83
**Tumor Characteristics**			
Grade			0.22
1	22 (12.7)	39 (14.9)	
2	48 (27.7)	64 (24.4)	
3	56 (32.4)	105 (40.1)	
Unknown	47 (27.2)	54 (20.6)	
Maximum extent on imaging			0.57
≤ 5 cm	19 (11.0)	38 (14.5)	
> 5 cm	79 (45.7)	115 (43.9)	
Unknown	75 (43.4)	109 (41.6)	
Clinical N stage			0.52
N0	164 (94.8)	246 (93.9)	
N1	5 (2.9)	5 (1.9)	
Unknown	4 (2.3)	11 (4.2)	
**Initial Treatment**			
Group			0.066
Resection only	5 (2.9)	18 (6.9)	
RT or chemo only	5 (2.9)	4 (1.5)	
Resection with neoadjuvant RT or chemo	27 (15.6)	57 (21.8)	
Resection with adjuvant RT or chemo	136 (78.6)	183 (69.8)	
Time to first treatment from diagnosis, days	30	36	**0.003**

**Table 5:** Multivariate analysis of predictors of overall survival for the subgroup of patients under and over 50 years of age

	Age < 50 (***N*** = 171)			Age ≥ 50 (***N*** = 261)		
	HR	(95% CI)	***P***-value*	HR	(95% CI)	***P***-value*
**Variable**						
Female sex	1.17	(0.63, 2.16)	0.62	0.51	(0.30, 0.87)	**0.013**
Age at diagnosis, per 10-year increase	0.96	(0.66, 1.39)	0.83	1.11	(0.83, 1.49)	0.48
Marital status			0.59			0.49
Married/significant other	(ref)			(ref)		
Single/separated/divorced	1.20	(0.63, 2.28)		0.82	(0.46, 1.45)	
Employment status			0.56			> 0.99
Full time/part time/student	(ref)			(ref)		
Unemployed/retired	1.36	(0.70, 2.62)		1.00	(0.55, 1.81)	
Unknown	1.50	(0.60, 3.73)		1.00	(0.43, 2.37)	
ADI WA decile, per 1-decile increase	0.97	(0.86, 1.09)	0.58	0.93	(0.84, 1.02)	0.12
**Tumor Characteristics**						
Grade			**0.016**			**0.004**
1	(ref)			(ref)		
2	1.99	(0.43, 9.27)		1.44	(0.57, 3.66)	
3	5.52	(1.24, 24.50)		2.87	(1.20, 6.86)	
Unknown	4.39	(0.97, 19.97)		0.86	(0.30, 2.44)	
Maximum extent on imaging			0.49			0.56
≤ 5 cm	(ref)			(ref)		
> 5 cm	1.42	(0.40, 5.00)		1.43	(0.64, 3.19)	
Unknown	1.89	(0.53, 6.68)		1.13	(0.50, 2.56)	
**Treatment**						
Time from diagnosis to first treatment, per 50% increase	0.95	(0.79, 1.15)	0.61	1.00	(0.85, 1.18)	0.97

*Wald test of HR = 1, without adjustment for multiple comparisons

## Discussion

This is the first study examining the association between OS and socio-economic factors in patients with STS treated at a single high-volume institution in the United States. Consistent with prior studies, patients with higher grade tumors or nodal disease had worse overall prognosis in our cohort. In the cohort of patients over 50, female gender was associated with better OS, which is possibly at least in part due to the overall longer life expectancy of women and other competing mortality factors in men [23].

Our initial analysis focusing on patient specific socio-economic factors, such as marital, employment and insurance status did not demonstrate a significant association with patients’ OS (Table 2). We additionally found that factors associated with patients’ permanent zip code, such as average income, rural vs urban location, distance from the treatment center, and ADI, were not associated with OS in this cohort. Finally, socioeconomic factors were not associated with OS in specific subgroups: patients treated with neoadjuvant therapy plus resection as well as patients above and below 50 years old (Table 5).

These findings are in contrast to the body of literature analyzing large populations treated in the USA and Europe, where multiple socio-economic factors, such as lower income, less than 10 years of education, and non-private insurance were associated with worse OS [14, 15, 19]. One explanation for this difference could be that these studies grouped together patients treated at many different institutions with variable STS experience and expertise. Previous studies have noted that while large nationwide databases have been instrumental in identifying broad trends, they have limitations when analyzing outcomes in rare malignancies, such as STS [24, 25].

Prior studies have proposed multiple hypotheses to explain these socio-economic disparities with worse cancer patient outcomes frequently associated with more limited access to care [26]. Patients with cancer who have lower education, lack private insurance, or identify as Black remain less likely to be referred to and treated at a high-volume center. This is especially problematic for rare cancers, such as STS, where recent evidence suggests that treatment at a high-volume institution may be associated with better OS [4, 5, 27, 28]. Interestingly, it has been suggested that in rare malignancies, such as pancreatic cancers, the effects of socio-economic factors, and marital status in particular, may be minimized when patients are treated in the setting of a clinical trial, suggesting that specialty care and clinical support may improve outcomes [29]. Cumulatively, these studies suggest that providing patients with access to high quality care may minimize some of the effects of pre-determined socio-economic factors. This may be especially important in patients with STS, who represent less than 1% of all malignancies and the treatment of whom requires an experienced, multi-specialty team approach. With average rate of 33 patients with STS evaluated per year, our center ranks in the top 1–2% high volume centers for treatment of STS in the United States [4, 5]. Given the previously documented improved outcomes in STS OS when patients were treated in centers such as ours, we are encouraged that no socio-economic factors associated with OS were identified.

This study has several important limitations that need to be considered. First, this is a single institution study and our results may not be generalizable to other institutions with different patient populations and treatment practice patterns. Second, this study carries the limitations of any retrospective study, with some data unavailable for review and variables that were not captured having potential influence on outcomes. Many patients were followed by a local oncologist after their initial therapy, limiting the median follow up of our cohort. This remains a limitation, despite the independent capture of patients’ date of death. This study is also limited by the fact that we did not have access to data regarding some important variables, such as margin status as well as race. Third, although most of the socio-economic data was patient specific and gathered from the EMR, other characteristics such as patient income, travel distance, and ADI were estimated based on the patient’s permanent ZIP code of residence. Thus, it may not completely capture the differences between patients living in the same geographic area. Next, patients treated at large academic institutions may be to a certain extent self-selected, and may represent a population with higher income and social support allowing them to travel to receive care. They or their family may also have higher health literacy urging them to seek a second opinion and pursue treatment at an academic institution. Lastly, our population may be enriched in patients referred from community providers due to need for more specialized or higher level of care.

## Conclusions

In this study, we were not able to identify socio-economic factors that are associated with changes in OS in patients with STS treated at a single high-volume academic institution. This is a hypothesis generating study, demonstrating that providing patients with soft tissue sarcomas access to a high volume treatment center and supporting them through their treatments may counteract some of the negative effect of their socio-economic circumstances. This strategy does present different challenges, but those may be more surmountable than changing deep seated socio-economic disparities.

## Supplementary Information

**Additional file 1 **: **Supplemental Table 1.** Patient age, income and distance by ZIP code presented in quartiles (*N* = 435). **Supplemental Table 2.** Relative correlation between different socioeconomic variables (Spearman rank correlation coefficients). **Supplemental Figure 1**. Consort diagram of patient population included in analysis. **Supplemental Figure 2.** Distribution of urban and rural counties at different Area Deprivation Indices (ADIs).

## Data Availability

The dataset analyzed during the current study is not publicly available since it contains protected personal information, but an anonymized set is available from the corresponding author on reasonable request.
