# Supplementary material for: Socio-economic factors do not affect overall survival in soft tissue sarcoma when patients treated at a single high-volume center
Source: BMC Cancer. 2021 May 27;21:620. doi: 10.1186/s12885-021-08352-z (PMC8157717; doi:10.1186/s12885-021-08352-z)
Supplement: Supplementary file 1 — Additional file 1 : Supplemental Table 1. Patient age, income and distance by ZIP code presented in quartiles (N = 435). Supplemental Table 2. Relative correlation between different socioeconomic variables (Spearman rank correlation coefficients). Supplemental Figure 1. Consort diagram of patient population included in analysis. Supplemental Figure 2. Distribution of urban and rural counties at different Area Deprivation Indices (ADIs). [file 12885_2021_8352_MOESM1_ESM.docx]

**Supplemental Table 1.**Patient age, income and distance by ZIP code presented in quartiles (N=435).

| **Demographics** | **No. (%) or**  **Mean ± SD** |
| --- | --- |
| Age at diagnosis, years | 52 ± 16 |
| 18-34 years | 138 (31.7) |
| 45-54 years | 99 (22.8) |
| 55-64 years | 95 (21.8) |
| 65+ years | 103 (23.7) |
| Average income from census, $ | 67,023 ± 20,458 |
| Q1 | 38 (8.9) |
| Q2 | 81 (19.0) |
| Q3 | 130 (30.4) |
| Q4 | 178 (41.7) |
| Mean distance by zip code (in-state only), miles | 71 ± 72 |
| Q1 | 239 (55.8) |
| Q2 | 110 (25.7) |
| Q3 | 50 (11.7) |
| Q4 | 29 (6.8) |

**Supplemental Table 2.** Relative correlation between different socioeconomic variables (Spearman rank correlation coefficients)

|  | **Average income** | **Education quantile** | **Mean Distance** | **ADI**  **Decile** | **Urban/Rural** |
| --- | --- | --- | --- | --- | --- |
| **Average income** | - | 0.65 | -0.58 | -0.81 | -0.38 |
| **Education quantile** | 0.65 | - | -0.54 | -0.81 | -0.24 |
| **Mean distance** | -0.58 | -0.54 | - | 0.70 | 0.38 |
| **ADI decile** | -0.81 | -0.81 | 0.70 | - | 0.30 |
| **Urban/rural** | -0.38 | -0.24 | 0.38 | 0.30 | - |

**Supplemental Figure 1**. Consort diagram of patient population included in analysis


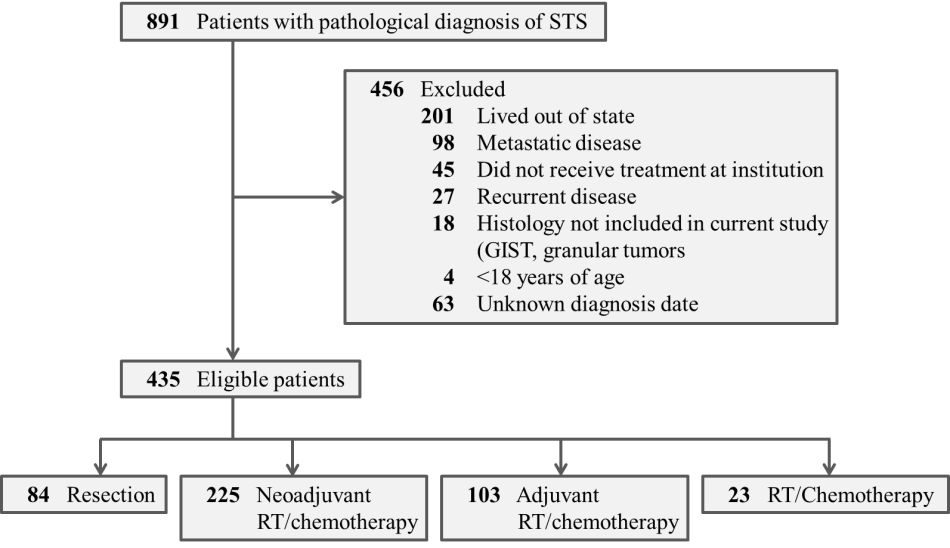


**Supplemental Figure 2.** Distribution of urban and rural counties at different Area Deprivation Indices (ADIs)


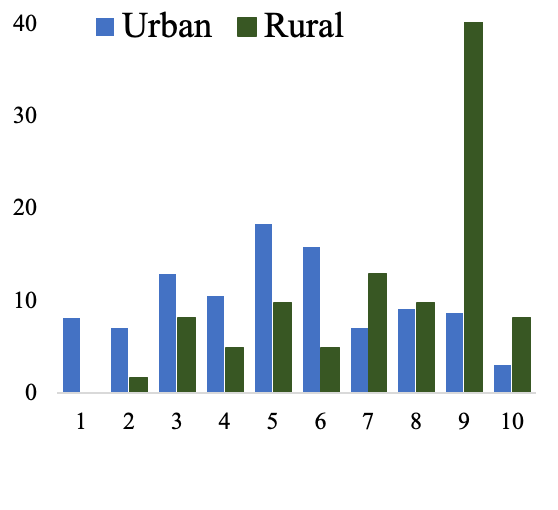


**ADI**

**Counties (%)**
